# Combined Strategies with Poly (ADP-Ribose) Polymerase (PARP) Inhibitors for the Treatment of Ovarian Cancer: A Literature Review

**DOI:** 10.3390/diagnostics9030087

**Published:** 2019-08-01

**Authors:** Stergios Boussios, Peeter Karihtala, Michele Moschetta, Afroditi Karathanasi, Agne Sadauskaite, Elie Rassy, Nicholas Pavlidis

**Affiliations:** 1 Acute Oncology Assessment Unit, Medway NHS Foundation Trust, Windmill Road, Gillingham ME7 5NY, Kent, UK; 2 AELIA Organization, 9th Km Thessaloniki-Thermi, 57001 Thessaloniki, Greece; 3 Department of Oncology and Radiotherapy, Medical Research Center Oulu, Oulu University Hospital and University of Oulu, P.O. Box 22, 90029 Oulu, Finland; 4 Drug Development Unit, Sarah Cannon Research Institute, 93 Harley Street, London W1G 6AD, UK; 5 Department of Pharmacy, Medway NHS Foundation Trust, Windmill Road, Gillingham ME7 5NY, Kent, UK; 6 Department of Cancer Medicine, Gustave Roussy Institut, 94805 Villejuif, France; 7 Department of Hematology-Oncology, Hotel Dieu de France University Hospital, Faculty of Medicine, Saint Joseph University, 166830 Beirut, Lebanon; 8 Medical School, University of Ioannina, Stavros Niarchou Avenue, 45110 Ioannina, Greece

**Keywords:** PARP inhibitors, ovarian cancer, *BRCA* mutations, homologous recombination deficiency, synthetic lethality, combination strategies

## Abstract

Poly (ADP-ribose) polymerase (PARP) inhibitors are the first clinically approved drugs designed to exploit synthetic lethality, and were first introduced as a cancer-targeting strategy in 2005. They have led to a major change in the treatment of advanced ovarian cancer, and altered the natural history of a disease with extreme genetic complexity and defective DNA repair via homologous recombination (HR) pathway. Furthermore, additional mechanisms apart from breast related cancer antigens 1 and 2 (*BRCA1/2*) mutations can also result in HR pathway alterations and consequently lead to a clinical benefit from PARP inhibitors. Novel combinations of PARP inhibitors with other anticancer therapies are challenging, and better understanding of PARP biology, DNA repair mechanisms, and PARP inhibitor mechanisms of action is crucial. It seems that PARP inhibitor and biologic agent combinations appear well tolerated and clinically effective in both *BRCA*-mutated and wild-type cancers. They target differing aberrant and exploitable pathways in ovarian cancer, and may induce greater DNA damage and HR deficiency. The input of immunotherapy in ovarian cancer is based on the observation that immunosuppressive microenvironments can affect tumour growth, metastasis, and even treatment resistance. Several biologic agents have been studied in combination with PARP inhibitors, including inhibitors of vascular endothelial growth factor (VEGF; bevacizumab, cediranib), and PD-1 or PD-L1 (durvalumab, pembrolizumab, nivolumab), anti-CTLA4 monoclonal antibodies (tremelimumab), mTOR-(vistusertib), AKT-(capivasertib), and PI3K inhibitors (buparlisib, alpelisib), as well as MEK 1/2, and WEE1 inhibitors (selumetinib and adavosertib, respectively). Olaparib and veliparib have also been combined with chemotherapy with the rationale of disrupting base excision repair via PARP inhibition. Olaparib has been investigated with carboplatin and paclitaxel, whereas veliparib has been tested additionally in combination with temozolomide vs. pegylated liposomal doxorubicin, as well as with oral cyclophosphamide, and topoisomerase inhibitors. However, overlapping myelosuppression observed with PARP inhibitor and chemotherapy combinations requires further investigation with dose escalation studies. In this review, we discuss multiple clinical trials that are underway examining the antitumor activity of such combination strategies.

## 1. Introduction

The striking sensitivity of breast related cancer antigens 1 and 2 (*BRCA1/2*) deficient tumour cells to poly (ADP-ribose) polymerase PARP inhibition have been demonstrated in 2005. Poly (ADP-ribose) polymerase (PARP) inhibitors have ultimately changed the way that patients with *BRCA*-related ovarian cancer are treated [1]. Olaparib, rucaparib, and niraparib have all obtained US Food and Drug Administration (FDA) and/or European Medicines Agency (EMA) approval in ovarian cancer in different settings. Veliparib does not yet have an approved label, and its use is being investigated mostly in combination with chemotherapy or targeted agents. All PARP inhibitors developed in epithelial ovarian cancers are PARP1/2 inhibitors, while olaparib and rucaparib also inhibit PARP3. In addition, rucaparib inhibits tankyrase-1, which is also included in the PARP family [2,3]. Recently, combined strategies with PARP inhibitors are also being actively investigated. Combinations of PARP inhibitors with drugs that inhibit homologous recombination (HR) may sensitize epithelial ovarian cancers with primary or secondary HR proficiency to PARP inhibitors and potentially expand their use beyond HR-deficient ovarian cancers. A test of DNA repair capability would accelerate the identification of cancers appropriate for PARP inhibition, taken that not all of the genes that affect DNA repair are known at the present time. An assay using a loss of heterozygosity to identify genomic scarring may be useful to predict PARP inhibitor response in *BRCA1/2* wild type ovarian cancers. Overall, it is not definitely established whether the promising results of preclinical studies will translate into improved clinical activity. As such, large studies with appropriate control arms and proper patient selection are extremely challenging for successful clinical implementation of novel PARP combination strategies, and could be achieved with international cooperation. This review will focus on the available evidence for PARP inhibitors combined separately with several agents, including anti-angiogenics, immune checkpoint inhibitors, phosphoinositide 3-kinase (PI3K), protein kinase B (AKT), mammalian target of rapamycin (mTOR), WEE1, mitogen-activated protein kinase (MEK), and cyclin dependent kinase (CDK) 4/6 inhibitors, as well as the traditional chemotherapy in the ovarian cancer. We also discuss ongoing clinical trials in this rapidly evolving area.

## 2. Mechanism of Action of PARP Inhibitors

PARPs are a family of 17 nucleoproteins with a common catalytic site that transfers an ADP-ribose group on a specific acceptor protein using NAD+ as a cofactor. Among PARP members, PARP1/2 participate in the complex landscape of DNA repair mechanism, chromatin modulation, mitosis, cell death, telomere length, and intracellular metabolism [2,4]. Single-strand DNA breaks in a normal cell are repaired by the base-excision repair pathway, in which PARP1/2 have a key role [5]. Inhibition of these proteins leads to single-strand breaks accumulation and, consequently to double strand DNA breaks and cytotoxicity. Unlike PARP2, PARP1 can also mediate the repair double-strand DNA breaks and damage to replication forks [6]. Therefore, inhibition of PARP1 is crucial for the impairment of these functions. In addition, PARP inhibitors may also function by trapping PARP1/2; PARP trapping occurs when the PARP enzyme is trapped on DNA by a PARP inhibitor, and prevents DNA repair, leading to cell death [3]. It has been demonstrated that PARP trapping is associated with PARP inhibitors cytotoxic activity.

For double-strand DNA breaks, cells heavily rely on HR repair mechanisms [7]. In cases of tumours that already lack this repair mechanism, PARP inhibition promotes cells to activate non-homologous end joining (NHEJ) repair. This is an error prone mechanism for repairing damaged DNA and as such unable to effectively repair DNA damage on a large scale [8]. Based on that, it has been proposed the hypothesis that tumours demonstrating HR deficiency are characterized by an improved response to PARP inhibition [9].

The most common clinical causes of HR deficiency are *BRCA1/2* germline mutations, identified in 9% and 8% of the ovarian cancers, respectively [10]. Germline *BRCA* mutated ovarian cancers have a distinct clinical behaviour characterized by younger age at diagnosis, visceral extent of the disease, higher response rates specifically to platinum chemotherapeutic agents, and sensitivity to PARP inhibitors [11].

However, alterations in *BRCA1/2* genes may also be the result of either somatic mutations, or epigenetic silencing in sporadic ovarian cancers. The clinical relevance is that extends activity of PARP inhibitors to a greater subset of sporadic ovarian cancer patients with HR deficiency. It has not yet been clarified whether the biological effects of harbouring somatic *BRCA1/2* mutations, a phenomenon termed as BRCAness, is identical to their germline counterparts. However, there are reports of patients with somatic *BRCA* mutations, who achieved longer progression-free survival (PFS) than wild-type cohorts, similarly to the population with germline *BRCA1/2* mutations. Nevertheless, overall survival (OS) was not affected significantly [11].

HR deficiency can result from epigenetic processes leading to silencing of HR genes including *BRCA1/2*. *BRCA* silencing may also occur by indirect mechanisms based on interactions of *BRCA* with other proteins participated in DNA repair. Epigenetic gene regulations at the promoter region CpG islands are highly dynamic, and the tumour microenvironment affects crucially methylation status. Next-generation sequencing of HGSOC tumours revealed that, independent of *BRCA1/2*, mutations in HR effectors, such as partner and localizer of *BRCA2* (*PALB2*), *RAD51*, *ATM*, *BRCA1*-interacting protein 1 (BRIP1), *BRCA1*-associated RING domain protein 1 (BARD1), and checkpoint kinase 2 (CHEK2) occurs in up to 20% of HGSOC [12]. Epigenetic silencing of *BRCA1* was mutually exclusive of *BRCA1/2* mutations, whilst survival was equal to the *BRCA1/2* wild type subset (41.5 months vs. 41.9 months). Overall, it seems that approximately 50% of HGSOC tumours are characterized by deficient or non-functional DNA repair pathways.

## 3. PARP Inhibition and Synthetic Lethality

Synthetic lethality was initially described nearly a century ago by geneticists as a situation in which two nonlethal defects combine and result in cell death [13]. In this context, a synthetic lethal interaction between *BRCA1* or *BRCA2* mutations and inhibition of PARP has been demonstrated [14]. This effect has also been demonstrated in a mouse xenograft model using *BRCA2*-deficient tumours cells. Treatment with a PARP inhibitor for five days, led to significant regression relative to *BRCA* wild-type tumours [15]. This provided the motivation to evaluate PARP inhibitors in clinical trials as single agents. Indeed, synthetic lethality approach may be more widely applicable in the treatment of sporadic cancers with impairments of the HR pathways or with BRCAness properties, providing an explanation of their sensitivity to PARP inhibitors. PARP inhibitors trap PARP1/2 on DNA, resulting in formation of toxic PARP–DNA complexes, known as “PARP trapping”, which affected PARP inhibitor development in two important ways [16]. Firstly, pathways other than HR may be essential for repairing the PARP–DNA complexes, supporting the treatment of tumours with defects in the FEN1, polymerase β, postreplication repair, and Fanconi anaemia pathways. Secondly, despite the fact that PARP inhibitors oppose the catalytic activity of PARP in general, there are remarkable differences in their abilities to trap PARP, based on the size and structure of each separate molecule. This finding may be related to the period of time that PARP is “trapped” onto the DNA, and provides an explanation of the significant differences in dosing among PARP inhibitors [6]. Veliparib shows a weaker PARP-trapping compared with niraparib, olaparib, and rucaparib. The ability to trap PARP1 may produce unacceptable toxicity in the case of therapeutic combination of PARP inhibitors with conventional doses of cytotoxic agents. PARP trapping is also the likely mechanism by which PARP inhibitors potentiate cytotoxic efficacy in HR-proficient cells [17].

## 4. PARP Inhibitors in Combination with Other Agents

The response of patients with recurrent ovarian cancer is being decreased following each subsequent line of therapy, particularly within platinum-resistant setting. Approximately 1/3 of patients with *BRCA1/2* mutations treated with single agent olaparib in this setting, respond to the treatment. A phase II study, enrolled a total of 298 patients with recurrent solid tumours who received olaparib with 400 mg twice daily. The tumour response rate was 26.2% (78 of 298; 95% CI, 21.3 to 31.6) overall and 31.1% (60 of 193; 95% CI, 24.6 to 38.1), in ovarian cancer patients. Stable disease for at least eight weeks was observed in 40% (95% CI, 33.4 to 47.7) of the ovarian cancer subgroup [18]. Combined strategies with PARP inhibitors are currently under investigation. Evaluation of additional tumorigenic pathways that may be affected by PARP inhibition as monotherapy or in combination with other cytotoxic and biologic agents to enhance antitumor efficacy is crucial [19]. Indeed, it has been proposed that there may be synergy between PARP inhibitors and other cell signaling pathway inhibitors, which represents a new treatment paradigm in ovarian cancer. *PARP1* has a role in HIF-1α stabilization and signaling mediated by nitric oxide and oxidative stress. Hypoxia induces the down-regulation of *BRCA1* expression, involved in numerous cellular pathways, including DNA repair, cell cycle checkpoint control, and transcriptional regulation. This down-regulation is associated with a functional decrease in HR activity in hypoxic cells. The target in the clinical practice is to reduce overlapping toxicities by optimizing dose and schedule, and to utilize the combinations to highly selected patients who would not otherwise benefit from single PARP inhibitors. In this direction, parameters for patients’ selection should include tumour type and molecular profiles, specifically for each unique PARP inhibitor-based combination. Several ongoing trials evaluate PARP combination strategies and provide insights into novel therapeutic options.

## 5. PARP Inhibitors and Antiangiogenic Agents

The first PARP inhibitor combinations to be studied were with antiangiogenic agents. The rationale for combination of PARP inhibitors with anti-angiogenic drugs has two purposes. It has been demonstrated that PARP inhibition decrease angiogenesis whereas, hypoxic state and vascular endothelial growth factor receptor 3 (VEGFR3) inhibitors induce down-regulation of HR repair proteins, such as BRCA1/2 and RAD51 [20,21,22], which potentiate PARP inhibitors sensitivity [23]. However, hypoxia is also associated with hypoxia inducible factor 1 alpha (*HIF1α*) up-regulation, and therefore resistance to angiogenesis inhibitors. Though, PARP1 is involved in *HIF1α* stabilization and consequently, inhibition of PARP may prevent *HIF1α* accumulation that leads to targeted hypoxic-induced apoptosis [24].

The combination of the antiangiogenic agent cediranib and olaparib vs. olaparib alone has been evaluated in a randomized, open label, phase II study (NCT01116648) [25,26]. Initially, the authors recorded preclinical synergy between olaparib and cediranib in the inhibition of ovarian cancer cell invasion and microvascular endothelial cell tube formation in vitro. Ninety women with platinum-sensitive, relapsed, HGSOC, or endometrioid ovarian cancer, stratified by *BRCA* mutation status (mutated vs. wild-type vs. unknown), were randomized to be treated with olaparib 400 mg twice daily or olaparib 200 mg twice daily plus cediranib 30 mg daily. Interim analysis revealed a significantly longer PFS of 17.7 months in the experimental arm compared with 9.0 months for patients treated with single agent olaparib (hazard ratio (HR) 0.42; *p* = 0.005). However, these results should be interpreted sceptically, due to small size of each subgroup. It would be interesting if the study design included an arm with single-agent cediranib to act as a comparator to the experimental arm. A post-hoc exploratory analysis demonstrated an increased activity of the combination vs. olaparib alone in the subset of patients with wild type or unknown *BRCA* status, with a statistically significant improvement in both median PFS (16.5 vs. 5.7 months (HR 0.32; *p* = 0.008)) and objective response rate (ORR; 76% vs. 32% (*p* = 0.006)). The authors proposed that this difference could be related to greater synergism between olaparib and cediranib in the setting of hypoxia, which potentially alters the DNA damage response gene expression [27]. Among patients with *BRCA* mutations, there was a lesser trend towards increased activity for the experimental arm, affected slighter the endpoints of PFS (19.4 vs. 16.5 months) and ORR (84% vs. 63% benefit). Overall, it seems that this combination may ideally be reserved for the patients with an intact HR repair phenotype. Finally, the very recently published updated analysis, demonstrated that OS was not statistically significantly different in the overall study population (44.2 vs. 33.3 months, HR 0.64; *p* = 0.11) [28]. Though, adverse events of grade III or higher were reported by 70% of patients, including mostly hypertension (41% vs. 0%), diarrhoea (23% vs. 0%), and fatigue (27% vs. 11%).

There are available additional ongoing trials for the evaluation of the combined treatment of PARP inhibitors with anti-angiogenics. The purpose of GY004 trial (NCT02446600) was to compare olaparib monotherapy vs. doublet therapy of olaparib and cediranib vs. standard platinum-based chemotherapy in patients with platinum-sensitive recurrent ovarian cancer [29]. Within the same setting, ICON 9 trial (NCT03278717) is examining maintenance therapy with the doublet of cediranib and olaparib, vs. single agent olaparib [30].

With regards to platinum-resistant disease, three phase II/III trials are currently in progress. COCOS study (NCT02502266) randomized patients to four treatment arms of each single agent olaparib and cediranib respectively, their combination, or the standard chemotherapy [31]. In OCTOVA study (NCT03117933), germline *BRCA* mutated participants were randomized to olaparib alone, olaparib with cediranib, or weekly paclitaxel, respectively [32]. Finally CONCERTO (NCT02889900) single-arm trial of olaparib/cediranib combination treatment enrolled only *BRCA* wild-type patients following at least three prior lines of chemotherapy [33].

The safety of the combination of olaparib with bevacizumab has been examined in a small phase I trial. A capsule formulation of olaparib at doses of 100, 200, and 400 mg twice daily was combined with 10 mg/kg bi-weekly bevacizumab [34]. Twelve patients were enrolled and the most frequently reported toxicities were grade I/II nausea and fatigue. The recommended phase II dose of olaparib was 400 mg twice daily. On the basis of these results, the Platine, Avastin, and OLAparib in the 1st Line trial (also known as PAOLA-1, NCT02477644) has been planned to evaluate the addition of olaparib vs. placebo to bevacizumab for patients treated in maintenance setting after upfront platinum chemotherapy [35]. AVANOVA (NCT02354131) is a phase II ongoing trial, comparing single-agent niraparib with combination niraparib-bevacizumab [36,37]. Ninety-four enrolled women with platinum-sensitive OC were assessed based on myChoice HR-deficient scores. The phase I component is a dose-escalation study for the evaluation of safety and tolerability of the bevacizumab–niraparib combination and recommendation of the phase II dose. The phase II component is a randomized three-arm study of niraparib vs. niraparib–bevacizumab combination vs. bevacizumab alone, evaluating treatment efficacy. Clinical trials of PARP inhibitors combined with antiangiogenic agents for treatment of ovarian cancer are resumed in Table 1.

## 6. PARP Inhibitors and Immune Checkpoint Inhibitors

An additional important issue of current research is whether, PARP inhibitors may enhance the response to immune checkpoint blockade or other immunotherapy approaches. It has been demonstrated over the past decade that the presence of tumour-infiltrating T cells within ovarian tumours is considered to be an indication of the host immune response to tumour antigens, and probably reflects the dynamic process of cancer immunoediting [39]. As such, tumour-infiltrating T cells are correlated with improved PFS and OS. Expanded analyses of immunological parameters, including antigen specificity of the infiltrating lymphocytes, and MHC expression by the tumour cells will potentially improve our understanding of the nature and role of tumour-infiltrating T cells in ovarian cancer. Immune checkpoint inhibitors prevent the suppression of cytotoxic immune cells, and promote tumour destruction by immune attack [40]. The therapeutic strategy of the combinations of PARP inhibitors with immunotherapies such as anti-cytotoxic T-lymphocyte-associated protein 4 (CTLA-4) and anti-programmed cell death protein 1 (PD-1)/programmed death-ligand 1 (PD-L1) has partly been based on the hypothesis that *BRCA1/2*, and wild-type *BRCA1/2* HR deficiency ovarian tumours display a higher neo-antigen load than HR-proficient cancers [41], which in turn produce more effective anti-tumour immune response [19]. In addition there is evidence that *BRCA* deficiency may induce a stimulator of interferon genes (STING)-dependent innate immune response [42], by inducing type I interferon and pro-inflammatory cytokine production [43]. Interestingly enough, preclinical models have also demonstrated that PARP inhibition inactivate glycogen synthase kinase 3 beta (*GSK3β*) and upregulate *PD-L1* in a dose-dependent manner. Consequently, T-cell activation is being suppressed, resulting in enhanced cancer cell apoptosis [44]. Among studies that investigate the utility of a combination of immune checkpoint blockade with PARP inhibitors, a TOPACIO trial and MEDIOLA study were recently presented at the European Society of Gynaecological Oncology (ESGO) congress 2018.

The phase I/II TOPACIO trial (NCT02657889) administers escalating doses of niraparib with pembrolizumab in a heavily pretreated platinum-resistant, or secondarily platinum-refractory cohort [45]. Based on dose finding in phase I, the recommended phase II dose of niraparib and pembrolizumab was 200 mg orally once daily and 200 mg intravenously three-weekly, respectively. Among 60 evaluable for initial response assessment patients, 64% had platinum-resistant, whereas 19% platinum-refractory, and 17% platinum-sensitive ovarian cancer, respectively [46]. In the entire population, the estimated ORR and disease control rate were 25% and 68%, respectively. Among enrolled patients, 77% were *BRCA* wild type, and 52% HR deficiency negative; even in these two subgroups, the ORR were 24% and 27%, respectively. This is suggestive of treatment efficacy in populations not typically responsive to single agent PARP inhibitors. On the other hand, the *BRCA1/2* mutant cohort of 11 patients, reached ORR and a disease control rate of 45% and 73%, respectively. In terms of safety concerns, preliminary data revealed adverse events compatible with those of single-agent strategies. The most common reported toxicities of grade 3 or more included anaemia (17%), fatigue (6%), and thrombocytopenia (3%) [47].

The phase I/II basket MEDIOLA trial (NCT02734004), evaluated the combination of olaparib and durvalumab in selected advanced solid cancers [48,49]. The phase I trial in patients with ovarian, triple-negative breast, cervical, or uterine cancer demonstrated reasonable tolerability of the combination, with no significant overlapping toxicities, accompanied by early evidence of efficacy [50]. In the phase II study among 32 patients with germline *BRCA1/2* mutant platinum-sensitive ovarian cancer, disease control rate at 12 weeks and ORR were 81% and 63%, respectively [48]. Within the 22 patients underwent one to two prior chemotherapies, the ORR was even more enhanced (68%). The most common reported adverse events of grade III or more were anaemia (12%) and increased lipase (9%), along with any-grade hypothyroidism (15%) and rash (12%) [48].

Apart from TOPACIO and MEDIOLA, additional early phase clinical trials evaluating PARP inhibitors in combination with immune checkpoint inhibitors are depicted in Table 2. The results of a small phase I (NCT02484404) dose-escalation study of durvalumab in combination with olaparib or cediranib have been reported in 2017 [50,51]. Doublet combination of durvalumab 10 mg/kg bi-weekly or 1500 mg four-weekly was investigated with escalating doses of either olaparib or cediranib, in patients with ovarian (*n* = 14), cervical, triple-negative breast cancer, and uterine leiomyosarcoma. Twelve out of the 26 enrolled patients received durvalumab and olaparib, while dose-limiting toxicities were not reported. The ORR was 17%; nevertheless, the majority of patients experienced some benefit yielding a disease control rate of 83%. Among 12 assessed patients treated with durvalumab plus cediranib, six attained partial response, and three stable diseases for at least four months, resulted in for a 50% ORR and a 75% disease control rate, respectively [50].

Several early ongoing studies are certainly encouraging for the novel combination strategy of olaparib with the *CTLA-4*-antagonist tremelimumab. NCT02571725 is a phase I/II study of olaparib at a dose of 300 mg twice a day (bis in die—BID), in combination with tremelimumab at a dose of 10 mg/kg monthly, for the treatment of *BRCA1/2* mutated recurrent ovarian cancer [52]. Similarly, a phase I/II trial (NCT02485990) is randomizing patients with persistent ovarian cancer and unknown *BRCA*-mutational status to tremelimumab alone or to the combination with olaparib, following one prior line of taxane-platinum-based chemotherapy [53]. The purpose of the study was to determine the dose of tremelimumab and olaparib that is potentially safe and effective in patients with persistent ovarian cancer including those with platinum-resistant disease. Finally, NCT02953457 is a phase I/II trial, recruiting patients with *BRCA* mutations and recurrent disease, investigating the combination of tremelimumab and olaparib, with the addition of durvalumab [54].

Three phase III studies in maintenance setting are in progress. FIRST (NCT03602859) was designed to assess platinum and the *PD-L1* inhibitor TSR-042, followed by niraparib and TSR-042 maintenance therapy, vs. standard platinum-based treatment followed by maintenance niraparib or placebo, as first-line treatment of advanced ovarian cancer [55]. ENGOT-ov46/AGO/DUO-O study (NCT03737643) evaluates the efficacy and safety of the standard approach of platinum-based chemotherapy and bevacizumab followed by maintenance bevacizumab either as monotherapy, or in combination with durvalumab, or in combination with durvalumab and olaparib [56]. Finally, ATHENA is a four-arm study (NCT03522246) that is currently investigating the combination of rucaparib with nivolumab, following response to front-line platinum-based chemotherapy [57].

## 7. PARP Inhibitors and Other Agents

Activation of the PI3K and RAS signal pathways is critical for the carcinogenesis and metastasis of HGSOC, and occurs in as much as 70% of all ovarian cancers [58]. Phosphatidylinositol-4, 5-bisphosphate 3-kinase catalytic subunit alpha (*PIK3CA*) gene amplification has also been associated with genomic instability, p53 mutation, and a lack of response to chemotherapy. Evidence from ovarian cancer cell lines and animal models revealed that activation of the PI3K/AKT pathway may lead to chemotherapy resistance. Suppression of apoptosis contributed to platinum and taxane resistance. The chemotherapy induced apoptosis was restored by specific PI3K inhibitors, such as wortmannin and LY294002 in vitro and in vivo [59]. The rationale for the combined approach of PI3K and PARP inhibitors was that PI3K inhibition lead to a downregulation of BRCA1/2 proteins, which increases the degree of HR repair deficiency [60]. In the absence of competent repair pathways, PARP inhibitors sensitize cells. With this regard, a phase I trial (NCT02208375) evaluated two different olaparib-containing PI3K combinations in patients with recurrent ovarian, endometrial, or triple negative breast cancer [61]. The novel agents targeted the PI3K pathway were the mTOR inhibitor vistusertib (AZD2014), and the AKT inhibitor capivasertib (AZD 5363). Preliminary results demonstrated that in the olaparib and AZD2014 arms, the ORR of patients with ovarian cancer was 20% [62]. Those treated with the combination of olaparib and AZD 5363 had a good tolerance, while several ovarian cancer patients achieved durable responses as well [63]. Interestingly enough, up to 95% of the participants were platinum-resistant, while 84% *BRCA* wild-type. Such therapeutic combinations of PARP inhibitors seem to be effective beyond *BRCA*-associated and/or HR-deficient cancers. Similarly, olaparib has been combined with AZD5363 in a study design that employed an accelerated intrapatient dose-escalation schema. The recommended phase II doses of AZD 5363 and olaparib were 640 mg twice daily two out of seven days, and olaparib 300 mg twice daily, respectively [64]. There is enough preclinical evidence that supports further investigation of the combination of olaparib with the pan-PI3K inhibitor buparlisib (BKM120) [65,66]. The PI3K pathway has been shown to be activated in a mouse model of *BRCA1*-mutated breast cancer and the combination of olaparib and BKM120 was synergistic and as such, related to improved efficacy, as compared to either agent alone [65]. Evaluation of this combination in vitro and in vivo revealed that, in vivo only, a significant suppression of tumour growth was observed. A similar result was seen in two patient-derived xenografts with sustained responses to the combination. In addition, PI3K p110α inhibition was found to render *BRCA1*-proficient tumours sensitive to the anti-cancer effects of olaparib using a murine breast cancer model [66]. The authors also reported that in vitro sensitivity to olaparib and veliparib could be achieved by PI3K pathway blockade. In this context was conducted a phase I trial of olaparib in combination with BKM120 (NCT01623349), enrolled 46 patients with ovarian and 24 with breast cancer [67,68]. From those with ovarian cancer, 22 patients (47.8%) achieved either partial response or stable disease. Among them, 17 women (38%) had platinum-resistant disease. There were detected 28 ovarian cancer patients with germ line *BRCA* mutations, who experienced partial response, and stable disease in 29% and 46%, respectively. At the same time, impressive treatment responses were also observed in the subset of eight *BRCA* wild-type patients, with partial response and stable disease of 12% and 62%, respectively. However, this combined therapeutic strategy requires attenuation of the BKM120 dose. Subsequently, a phase I trial evaluated the combination of olaparib with alpelisib, which is a more specific PI3K-alpha inhibitor [69]. The demonstrated ORR in patients with advanced ovarian cancer, who were mostly platinum-resistant, was 36%. It seems that the benefit is not related to the germline DNA damage response gene mutational status, similarly to the outcome of the previous study with the combination of olaparib with BKM120 [68]. This preliminary clinical evidence of synergism between olaparib and alpelisib warrants further investigation. In addition, estimation of the efficacy of PI3K/PARP-inhibitor combinations vs. PARP inhibitors monotherapies in different settings of recurrent ovarian cancer is crucial. Indeed, OReO/ENGOTOv-38 is an ongoing trial that aims to assess efficacy and safety of maintenance re-treatment with olaparib in patients who relapse whilst on olaparib maintenance but retain platinum sensitivity.

WEE1 inhibitors may also be effectively combined with PARP inhibitors; the underlying synergy dependent on the PARP trapping ability of the PARP inhibitor [70]. Both replication stress and nucleotide resource depletion are induced when PARP inhibitors are combined with either WEE1 or Rad3-related protein (ATR) inhibitors. Additional DNA damage response inhibitors that could potentially been combined with PARP include ATM, checkpoint kinases 1 and 2 (CHK1/2), DNA-dependent protein kinase (DNA-PK), and DNA polymerase θ (POLθ) and currently are in clinical development [71]. Further research with dose escalation studies and evaluation of sequence of these therapies is required, in order to be avoided overlapping myelosuppression.

Additional promising results of preclinical drug interaction studies are available that would be translated into improved clinical activity. With this regard, it has been demonstrated that MEK inhibitors decrease HR repair gene expression, and there is synergistic activity with PARP inhibitors in vitro and in vivo in tumours with mutant RAS [72]. Indeed, KRAS and NRAS mutant tumours and to a lesser extent BRAF mutant tumours are highly sensitive to combinations of PARP with MEK/ERK inhibitors in vitro, whereas for KRAS mutant models this evidence is available in vivo. Both in vitro and in vivo data argue that a MEK and PARP inhibitors combination has the potential to induce cell death and increase the magnitude, duration, and spectrum of PARP inhibitors activity. Resistance to PARP inhibitors in cell lines selected for resistance in vitro as well as in cells recultured from PARP inhibitors treated tumours in vivo was reversed by MEK inhibitors. This was suggestive of activity of PARP and MEK inhibitors combination in patients who have failed PARP inhibition. Based on that, NCT03162627, a phase I/II trial of selumetinib and olaparib in patients with *RAS*-altered solid tumours, and those who experience disease recurrence during prior PARP inhibitor treatment is currently recruiting patients [73]. Furthermore, bromodomain and extra-terminal motif (BET) inhibitors suppress DNA damage response genes, such as DNA topoisomerase 2-binding protein 1 (TOPBP1), and WEE1 and their combination with PARP inhibitors is an area of early phase research [74].

Therapeutic synergy for combined PARP and CDK4/6 inhibition has recently been demonstrated. It seems that MYC status represent a determinant of sensitivity to this combined treatment in ovarian cancer cells both in vitro and in vivo. Palbociclib induces HR deficiency through downregulation of MYC-regulated HR pathway genes, which lead to synthetic lethality with olaparib [75]. Early randomized studies of olaparib in combination with additional cell signaling pathway inhibitors are depicted in Table 3.

## 8. PARP Inhibitors Combined with Chemotherapy

Combinations of PARP inhibitors with conventional chemotherapy agents that induce DNA strand breaks is based on the fact that PARP inhibitors block base excision repair, and consequently potentiate cytotoxics’ efficacy [78,79,80,81]. Taken that inhibition of PARP in normal cells abrogates an important mechanism of DNA repair in these cells, chemotherapy induced myelosuppression is enhanced. Based on that, a major concern for this combined therapeutic approach is the high risk of overlapping myelotoxicity [82]. Consequently, dose modification of both regimens is recommended.

A phase I, 3 + 3 dose escalation study investigated the combination of carboplatin and olaparib, in a cohort of 45 women with either ovarian or breast cancer and a germline *BRCA* mutation [80]. It was demonstrated that olaparib 400 mg twice daily for 14 days combined with standard three-weekly carboplatin area under the curve (AUC) 5 was well tolerated and effective. Among ovarian patients, 44% reached partial response, whereas 41% attained stable disease between three and more than 25 months.

Another phase I study (NCT00516724) investigated the combination of olaparib with either weekly paclitaxel, three-weekly carboplatin, or with the doublet chemotherapy, in patients with several advanced solid tumours refractory to standard therapies, including ovarian cancer [79,83]. However, those treated with continuously daily olaparib in combination with carboplatin and paclitaxel exacerbated hematologic toxicities leading to schedule delays. Tolerability has been improved with intermittent olaparib, whereas efficacy was highest in patients with *BRCA1/2* mutations. The study identified two tolerable olaparib treatment schedules for further development.

Study 41 (NCT01081951) is an open label, randomized 1:1 phase II study that stratified patients by the number of platinum-based treatments and platinum-free interval [78,84]. In this setting of platinum-sensitive recurrent ovarian cancer, the experimental arm of olaparib with carboplatin and paclitaxel achieved a significant improvement in PFS as compared to the chemotherapy arm (HR 0.51; 95% CI 0.34–0.77; *p* = 0.0012; median = 12.2 vs. 9.6 months). The treatment benefit seemed to derive mostly from the maintenance olaparib monotherapy phase, and it was greater in patients with *BRCA1/2* mutations. Nevertheless, the ORR was similar between treatment arms (64% vs. 58%). This might have been compromised by an imbalance in early censoring. Overall, treatment tolerance was reasonable and manageable. Serious adverse events were reported in 15% of patients in the experimental group, and in 21% of those in the chemotherapy alone group.

As far as veliparib is concerned, its combination with chemotherapy has been investigated in both chemo-naïve patients and those with recurrent disease. GOG-3005, (NCT02470585) is an active, randomized, three-arm trial evaluating carboplatin and paclitaxel vs. chemotherapy combined with veliparib vs. the combination followed by veliparib maintenance therapy in the first-line treatment of ovarian cancer [85]. Veliparib is also currently being evaluated in combination with temozolomide vs. pegylated liposomal doxorubicin in a randomized phase II study (NCT01113957) in patients with recurrent HGSOC [86]. Primary end point of the study was the ORR based on radiological and biochemical evidence. The accrual has been completed and results are awaiting.

However, some of the trials of the combinations of PARP inhibitors with conventional chemotherapy have failed to demonstrate efficacy. In the setting of recurrent ovarian cancer, a randomized phase II trial (NCT01306032) was conducted to determine the ORR of veliparib in combination with cyclophosphamide, compared with cyclophosphamide alone in patients with pretreated *BRCA*-mutant ovarian cancer or those with pretreated primary peritoneal, fallopian tube, or HGSOC [87,88]. The study demonstrated that addition of 60 mg daily veliparib to 50 mg daily oral cyclophosphamide did not improve either the ORR, or the median PFS, as compared to oral cyclophosphamide monotherapy. The relatively small sample set may have affected the outcome of the trial. PFS of patients treated with the combination, stratifying by *BRCA*-mutant status was analysed. *BRCA* status from tumour exome analysis exhibited a slight trend toward an effect in patients treated with the combination (*p* = 0.22), indicating a role in the prognosis. Furthermore, a dose of veliparib employed in the study was below the standard 250–400 mg BID doses used so far. Indeed, higher doses of veliparib in combination with cyclophosphamide may have affected the efficacy. Finally, the presence of DNA repair defects did not predict for response to either cyclophosphamide or the combination of veliparib and cyclophosphamide.

The combination of topoisomerase inhibitors with PARP inhibitors in ovarian cancer has been investigated as well. In 2012, a phase I clinical trial reported an ORR of 32% (6/9 patients) for the combination of olaparib with topotecan in solid tumours. However, the investigators did not recommend the use of this therapeutic option based on increased adverse effects, mostly neutropenia [89]. The study also demonstrated decreased bioavailability of olaparib with concomitant topotecan treatment probably, taken the overlap in drug efflux action. As such, an altered dosing schedule could prove to be more effective. Pre-clinical in vitro synergy of topotecan and veliparib has been demonstrated and occurred at veliparib combinations below those needed to kill HR deficient cells. The safety of this combination was also investigated in a phase I/II clinical trial in the setting of recurrent ovarian cancer with *BRCA1/2* wild-type or unknown mutational status (NCT01690598) [90]. Among 27 enrolled patients in both phase I and phase II parts, 10 (37%) achieved stability or even response of the disease. Median PFS was 2.8 months (95% CI: 2.6–3.6), whereas median OS reached 7.1 months (95% CI: 4.8–10.8). The interpretation of this modest efficacy should be made, taken into account the negative prognostic factors of *BRCA1/2* wild-type/unknown status, and platinum resistance/refractory disease in study’s population.

Combination trials with PARP inhibitors and chemotherapy are reported in Table 4.

## 9. Conclusions and Future Perspectives

The path forward for PARP inhibitor treatment in ovarian cancer is multifaceted. More research is warranted in order to deepen our understanding of DNA repair mechanisms, cancer biology, and targeted therapies. Resistance to PARP inhibitors also need to be further explored. Tumour biopsies on disease progression would be critical for the identification of mechanisms of drug resistance. Therapeutically, combinations of PARP inhibitors with drugs that inhibit HR might be an effective approach to sensitize ovarian cancers with de novo or acquired HR proficiency to PARP inhibitors. However, further studies are required in order to better define predictive biomarkers beyond *BRCA* mutations and HR deficiency status, and to facilitate patient stratification for combined therapy. It is also challenging evaluation of the risk of adverse events, specifically in the combination strategies. With this regard, optimization of the treatment dose in order to be maximized the overall risk-benefit profile of a given combination, would be based on dose escalation studies that can however be expensive. Finally, high cost of therapy remains target for improvement of PARP inhibitors, both as single agents and in combination with other drugs. If we manage to address these issues, PARP inhibitors would move into the forefront of ovarian cancer treatment.

## Figures and Tables

**Table 1:** Ongoing combination trials with PARP inhibitors and antiangiogenic agents (www.clinicaltrials.gov).

Agent	Trial/References	Phase	Planned *n*	Combination	Population	Status
Olaparib	NCT01116648/[25,26]	II	162	Arm (1): Olaparib + cediranib Arm (2): Olaparib alone	- Relapsed platinum sensitive recurrent HGSOC/HGEOC - Unselected for *BRCA* mutation status	Active, not recruiting
	NCT02446600 (NRG-GY004)/[29]	III	549	Arm (1): Olaparib alone Arm (2): Olaparib + cediranib Arm (3): Physician choice chemotherapy	- Recurrent, platinum sensitive OC - Germline *BRCA1/2* - Any *BRCA* mutation status	Active, not recruiting
	NCT03278717 (ICON 9)/[30]	III	618	Arm (1): Olaparib + cediranib Arm (2): Cediranib + placebo (maintenance therapy)	- PR or CR to platinum chemotherapy - Any *BRCA* mutation status	Recruiting
	NCT02502266 (COCOS)/[31]	II, III	680	Arm (1): Olaparib alone Arm (2): Cediranib alone Arm (3): Olaparib + cediranib Arm (4): Physician choice chemotherapy	- Platinum-resistant or–refractory HGSOC - Germline *BRCA1/2*	Recruiting
	NCT03117933 (OCTOVA)/[32]	II	138	Arm (1): Paclitaxel alone Arm (2): Olaparib alone Arm (3): Olaparib + cediranib	- Relapsed platinum resistant OC - Stratification for prior PARP use, prior anti-angiogenic use and *BRCA* status	Recruiting
	NCT02889900 (CONCERTO)/[33]	IIb	62	Single arm: Olaparib + cediranib	- Relapsed HGSOC/HGEOC - No germline mutation in *BRCA1/2*	Active, not recruiting
	NCT02477644 (PAOLA-1)/[35]	III	612	Arm (1): Platinum/taxane/bev, followed by bev maintenance Arm (2): Platinum/taxane/bev, followed by bev/olaparib maintenance (maintenance therapy)	- Newly-diagnosed OC - PR or CR to platinum chemotherapy with bev - Planned bev maintenance - Any *BRCA* mutation status	Recruiting
	NCT02681237/[38]	II	34	Single arm: Olaparib + cediranib	- Relapsed HGSOC/HGEOC with progression on PARP inhibitor	Active, not recruiting
Niraparib	NCT02354131 (AVANOVA)/[36,37]	III	108	Single arm: Niraparib + bev	- Platinum-sensitive OC - HRD	Active, not recruiting

PARP: Poly (ADP-ribose) polymerase; Bev: Bevacizumab; OC: Ovarian cancer; PR: Partial response; CR: Complete response; BRCA: Breast related cancer antigens; HGSOC: High-grade serous ovarian cancer; HGEOC: High-grade endometrioid ovarian cancer; HRD: Homologous recombination deficient.

**Table 2:** Ongoing combination trials with PARP inhibitors and immunotherapy (www.clinicaltrials.gov).

Agent	Trial/References	Phase	Planned *n*	Combination	Population	Status
Olaparib	NCT02734004 (MEDIOLA)/[48,49]	I/II	427	Single arm: Olaparib + durvalumab	Basket study in: - gBRCAmut OC, - gBRCAmut HER2(–) breast cancer, - Relapsed platinum-sensitive SCLC, - Metastatic or relapsed gastric cancer	Recruiting
	NCT02484404/[50,51]	I/II	384	Arm (1): Durvalumab + Olaparib Arm (2): Durvalumab + Cediranib Arm (3): Durvalumab + Olaparib + Cediranib	Basket study in previously treated: - Platinum resistant OC, - <3 prior lines, *gBRCAmut* TNBC, - ≥2 prior lines NSCLC, - ≥2 prior lines SCLC, - mCRPC, - 3rd line microsatellite stable colorectal cancer	Recruiting
	NCT02571725/[52]	I/II	50	Single arm: Olaparib + tremelimumab	- Recurrent *gBRCAmut* OC	Recruiting
	NCT02485990/[53]	I/II	68	Arm (1): Tremelimumab alone Arm (2): Tremelimumab + olaparib Arm (3): Tremelimumab + olaparib [Dose will be determined during the Arm (2)]	- Recurrent or persistent OC	Recruiting
	NCT02953457/[54]	I/II	39	Durvalumab + tremelimumab + olaparib	- Recurrent platinum-sensitive or resistant or refractory OC - *BRCA1/2* mutation (both germline and sporadic)	Recruiting
	NCT03737643 (ENGOT-ov46/AGO/DUO-O)/[56]	III	1056	Three double-blind treatment arms cohort for patients with no *BRCA* mutations: Arm (1): Platinum-based chemotherapy + bev and durvalumab placebo, followed by maintenance bev, durvalumab placebo, and olaparib placebo Arm (2): Platinum-based chemotherapy + bev and durvalumab, followed by maintenance bev, durvalumab and olaparib placebo Arm (3): Platinum-based chemotherapy + bev and durvalumab, followed by maintenance bev, durvalumab and olaparib Single open label arm cohort for patients with *BRCA* mutation: Platinum-based chemotherapy + bev and durvalumab, followed by maintenance bev, durvalumab and olaparib	- Newly diagnosed advanced (FIGO stage III-IV) OC	Recruiting
Niraparib	NCT02657889 (TOPACIO/ Keynote-162)/[45,46]	I/II	121	Single arm: Pembrolizumab + niraparib	Basket study in: - *HER2*(–) breast cancer, - Recurrent platinum-resistant OC	Active, not recruiting
	NCT03602859 (FIRST trial)/[55]	III	912	Arm (1): Chemotherapy + TSR-042 placebo, followed by maintenance treatment of niraparib placebo and TSR-042 placebo Arm (2): Chemotherapy treatment + TSR-042 placebo, followed by maintenance treatment of niraparib and TSR-042 placebo Arm (3): Chemotherapy treatment + TSR-042, followed by maintenance treatment of niraparib and TSR-042	- Newly diagnosed advanced (FIGO stage III-IV) OC (first-Line Treatment)	Recruiting
Rucaparib	NCT03522246 (ATHENA)/[57]	III	1012	Arm (1): Nivolumab + Rucaparib Arm (2): Placebo + Rucaparib Arm (3): Nivolumab + Placebo Arm (4): Placebo + Placebo	- Newly diagnosed advanced (FIGO stage III-IV) OC - Completed first-line platinum-based chemotherapy and surgery with a response (maintenance treatment)	Recruiting

PARP: Poly (ADP-ribose) polymerase; OC: Ovarian cancer; BRCA: Breast related cancer antigens; gBRCAmut: Germline BRCA mutated; TNBC: Triple negative breast cancer; NSCLC: Non-small cell lung cancer; SCLC: Small cell lung cancer; mCRPC: Metastatic castration-resistant prostate cancer; HER2: Human epidermal growth factor receptor 2; Bev: Bevacizumab; FIGO: International Federation of Gynaecology and Obstetrics.

**Table 3:** Ongoing combination trials with olaparib and other cell signaling pathway inhibitors (www.clinicaltrials.gov).

Trial/Reference	Phase	Planned n	Signaling Pathway	Combination	Population	Status
NCT02208375/[61]	Ib	159	PI3K/AKT/mTOR	Arm (1): Olaparib + AZD2014 (continuous dosing), Arm (2): Olaparib + AZD2014 (intermittent dosing), Arm (3): Olaparib + AZD5363 (intermittent dosing)	- Locally advanced recurrent endometrial adenocarcinoma - Recurrent HGSOC - *gBRCAmut* OC of any histology	Active, not recruiting
NCT01623349/[67]	I	118	PI3K/AKT/mTOR	Arm (1): Olaparib + BKM120, Arm (2): Olaparib + alpelisib	- Recurrent TNBC or HGSOC - Prior therapy for HGSOC must have included a first-line platinum-based regimen	Active, not recruiting
NCT03162627/[73]	I/II	90	Wee1 inhibition	Single arm: Olaparib + selumetinib	- *RAS*-altered cancers (*KRAS*, *NRAS*, *NF1*, *HRAS*, and *BRAF*), - PARP-inhibitor resistant OC	Recruiting
NCT02576444 (OLAPCO)/[76]	II	64	PI3K/AKT/mTOR	Arm (1): Olaparib alone, Arm (2): Olaparib + capivasertib, Arm (3): Olaparib + adavosertib, Arm (4): Olaparib + Vistusertib	Advanced solid tumours that harbour: - Mutation in *HDR* genes, - *PTEN*, *PIK3CA*, *AKT*, or *ARID1A* mutations, - Either *TP53* or *KRAS* mutations or mutations in *KRAS* and *TP53*, - Mutations in *HDR* genes, including *ATM*, *CHK2*, *APOBEC*, *MRE11* complex	Recruiting
NCT02338622 (COMPAKT)/[77]	I	58	PI3K/AKT/mTOR	Single arm: Olaparib + capivasertib	Advanced solid tumours - TNBC, - CRPC, - HGSOC, - Tumours with somatic mutations or other aberrations known to result in a hyperactivated *PI3K-AKT* pathway, - *gBRCAmut* cancers	Unknown

HDR: Homology-directed repair; PTEN: Phosphatase and tensin homolog; PIK3CA: Phosphatidylinositol-4,5-bisphosphate 3-kinase catalytic subunit alpha; AKT: Protein kinase B; ARID1A: AT-rich interaction domain 1A; KRAS: Kirsten rat sarcoma 2 viral oncogene homolog; CHK2: Checkpoint kinase 2; APOBEC: Apolipoprotein B mRNA editing catalytic polypeptide-like; MRE11: Meiotic recombination 11 homolog A; TNBC: Triple negative breast cancer; CRPC: Castration-resistant prostate cancer; HGSOC: High-grade serous ovarian cancer; gBRCAmut: Germline BRCA mutated; NRAS: Neuroblastoma ras viral oncogene homolog; NF1: Neurofibromatosis type 1; HRAS: Harvey rat sarcoma viral oncogene homolog; BRAF: V-raf murine sarcoma viral oncogene homologue B1; PARP: Poly (ADP-ribose) polymerase; OC: Ovarian cancer; HGSOC: High-grade serous ovarian cancer; TNBC: Triple negative breast cancer.

**Table 4:** Ongoing combination trials with PARP inhibitors and chemotherapy (www.clinicaltrials.gov).

Agent	Trial/References	Phase	Planned n	Combination	Population	Status
Olaparib	NCT01081951 (Study 41)/[78,84]	II	162	Arm (1): Olaparib (200 mg BID, D1–10/21) + paclitaxel (175mg/m^2^, D1/21) + carboplatin (AUC4, D1/21), followed by olaparib maintenance Arm (2): Carboplatin (AUC6, D1/21) + paclitaxel (175 mg/m^2^, D1/21)	- ≤3 prior lines οf platinum-based treatments, - Platinum sensitive recurrent HGSOC (both germline *BRCA* and sporadic)	Active, not recruiting
	NCT00516724/[79,83]	I	189	Arm (1): Olaparib + carboplatin Arm (2): Olaparib + paclitaxel Arm (3): Olaparib + carboplatin and paclitaxel	- >2 prior lines οf platinum-based treatments	Active, not recruiting
	NCT01445418/[91]	I/Ib	45	3 + 3 dose escalation incorporated continuous daily or intermittent olaparib capsules at doses of 100 to 400 mg every 12 h with carboplatin (AUC3–5, D1/21), followed by olaparib maintenance of olaparib	- gBRCAmut - No prior PARP inhibitors	Completed
Veliparib	NCT02470585 (GOG 3005)/[85]	III	1140	Arm (1): Carboplatin + paclitaxel followed by placebo maintenance Arm (2): Carboplatin + paclitaxel + veliparib followed by placebo maintenance Arm (3): Carboplatin + paclitaxel + veliparib followed by veliparib maintenance	- Advanced HGSOC - Any *BRCA* mutation	Active, not recruiting
	NCT01113957/[86]	II	168	Arm (1): Veliparib and temozolomide Arm (2): PLD	- Recurrent HGSOC - Both germline *BRCA* and sporadic	Completed, waiting results
	NCT01306032/[87,88]	II	124	Arm (1): Oral cyclophosphamide (50 mg OD) + veliparib (60 mg OD) Arm (2): Oral cyclophosphamide (50 mg OD)	- Recurrent HGSOC - Both germline *BRCA* and sporadic	Completed
	NCT00516438 Samol, J.; et al./[89]	I	21	Arm (1): Topotecan + olaparib (dose escalation) Arm (2): Olaparib (dose escalation)	- Advanced solid tumours	Completed
	NCT01690598/[90]	I/II	27	Veliparib BID on days 1–3, 7–9, and 14–16 + Topotecan 2 mg/m² on days 2, 8, and 15	- Recurrent HGSOC - *BRCA* negative or unknown	Completed

PARP: Poly (ADP-ribose) polymerase; BID: Twice a day (bis in die); D: Day; AUC: Area under the curve; HGSOC: High-grade serous ovarian cancer; BRCA: Breast related cancer antigens; gBRCAmut: Germline BRCA mutated; PLD: Pegylated liposomal doxorubicin; OD: Once a day (omne in die).
